# A rotationally focused flow (RFF) microfluidic biosensor by density difference for early-stage detectable diagnosis

**DOI:** 10.1038/s41598-021-88647-0

**Published:** 2021-04-29

**Authors:** Noori Kim, Kyungsup Han, Pei-Chen Su, Insup Kim, Yong-Jin Yoon

**Affiliations:** 1 Department of Electrical and Electronic Engineering, Newcastle University in Singapore, 172A Ang Mo Kio Avenue 8, 05-01 SIT@NYP Building, Singapore, 567739 Singapore; 2 School of Mechanical and Aerospace Engineering, Nanyang Technological University (NTU), Singapore, 639798 Singapore; 3 Department of Mechanical Engineering, Korea Advanced Institute of Science and Technology (KAIST), Daejeon, 34141 Korea

**Keywords:** Mechanical engineering, Sensors and biosensors

## Abstract

Label-free optical biosensors have received tremendous attention in point-of-care testing, especially in the emerging pandemic, COVID-19, since they advance toward early-detection, rapid, real-time, ease-of-use, and low-cost paradigms. Protein biomarkers testings require less sample modification process compared to nucleic-acid biomarkers’. However, challenges always are in detecting low-concentration for early-stage diagnosis. Here we present a Rotationally Focused Flow (RFF) method to enhance sensitivity(wavelength shift) of label-free optical sensors by increasing the detection probability of protein-based molecules. The RFF is structured by adding a less-dense fluid to focus the target-fluid in a T-shaped microchannel. It is integrated with label-free silicon microring resonators interacting with biotin-streptavidin. The suggested mechanism has demonstrated 0.19 fM concentration detection along with a significant magnitudes sensitivity enhancement compared to single flow methods. Verified by both CFD simulations and fluorescent flow-experiments, this study provides a promising proof-of-concept platform for next-generation lab-on-a-chip bioanalytics such as ultrafast and early-detection of COVID-19.

## Introduction

Corona Virus Disease 2019, aka COVID-19, is an epidemic caused by severe acute respiratory syndrome coronavirus 2 (SARS-CoV2). On 11th March 2020, the COVID-19 was declared a pandemic by the World Health Organization (WHO)^1^. The COVID-19 term has been used colloquially to indicate the SARS-CoV2 virus, respiratory illness caused by SARS-CoV2, and the coronavirus pandemic based on the context.

The respiratory tract infection caused by the COVID-19 was firstly reported from Wuhan, Hubei Province, the People’s Republic of China in December 2019^2^. Since the onset of the outbreak, this new pandemic pathogen has spread readily and rapidly over the world. In general, a virulent virus is not transmissible. However, the SARS-CoV2 is not much destructive compared to other epidemic viruses (i.e., Ebola or different types of coronavirus). Clinical manifestations of the COVID-19 are broad as well as the degree of severity from people in asymptomatic to fatality. It can invade the human body furtively, as reported by many studies regarding no-symptom infections and aerosol transmissions^3–6^.

Biosensors have been critical elements in developing the COVID-19 testing kits, vaccines, and antiviral drugs to diagnose, prevent, and treat the disease. They have been widely used as immuno-sensors for the detection of target biomolecules in body fluids because they offer several advantages such as rapid and continuous measurement, high sensitivity and lower specificity, and fewer reagents usage^7–9^. Among different types of biosensors^10–14^, label-free optical biosensors have gained significant attractions because the target molecule can be detected as its natural form without any alteration. They are relatively inexpensive, easy to handle and allow quantitative and real-time detection^15–18^. However, the detection limit of a label-free optical sensor may not meet the requirement in a clinical setting. In the low concentration range, the chance of false-positive is extremely low, hence the sensitivity of sensors can suffer^19,20^. Therefore, developing a highly sensitive sensor system with a broad dynamic range is critical to detect clinically relevant protein biomarkers^21,22^.

As one of the efforts to improve the sensitivity and the detection limit of biosensors is enhancing binding kinetics between analytes and immobilised ligands on the surface of biosensors by reducing the boundary layer thickness^23–25^. Typically, convective transportation is dominant in a microfluidic device due to its high Péclet number (Pe) over 10^3^ based on an extremely narrow width of the microchannel and low diffusivity of analyte solutions^26,27^. In this case, binding kinetics can be effectively augmented by increasing initial reactive flux, which is directly enhanced by the reduction of boundary layer thickness^28–30^. Reducing the height, however, may induce an excessively high-pressure drop (i.e., Darcy–Weisbach equation for the rectangular-shaped channel, $P\propto {h}^{-2}$)^26,27^ that could cause breakage of the bonding between microfluidic channel above a particular bonding strength^31^. Therefore, it is desirable to develop a straightforward microfluidic approach that can be ubiquitous with any sensor platforms and induce enhanced performances for target applications.

One of the SARS-CoV2′s genetic and structural features is a protein on its surface, which would give a genetic clue of contagiousness to the human body^32^. Recent proteomics reported^27^ potential biomarkers, including proteins, to indicate severity levels of COVID-19 symptom. Although polymerase chain reaction (PCR) based diagnostics have benefits in test scalability, they provide limited information on the severity of the disease, nor the course of illness^33^. Also, the viral RNA preparation time in real-time PCR (aka RT-PCR) process may affect the diagnostic accuracy as well as slow down the testing time. In a general laboratory setting, biomarker isolation and simple preparation process with protein-based biomarkers are more uncomplicated and comfortable compared to a nucleic acid (DNA/RNA) or cell-based biomarkers^34^. The protein-based biomarkers can help in early-stage diagnostics requiring minimum modification of samples, such as no amplification. To address the limitations with the RT-PCR method, Seo et al. developed a biosensor that detects the SARS-CoV-2 antigen protein using a field-effect^35^. However, their approach relies on the sensor system that they designed, a graphene-based biosensing device, and also requires longer incubation time (i.e., 4 h)^34^.

Also, various methods integrated with a microfluidic channel for streptavidin–biotin complex detection have been investigated^36–38^. Chen, Ting-Yang, et al., have developed an electrical detection with thin film transistor-based biosensor integrated with a microfluidic channel by monitoring the current response of biotin and streptavidin. Yang, Haoyue, and Toshiya Sakata also have employed a microfluidic system able to monitoring the electrical signals induced by the change in pH resulting from the biotin-streptavidin interaction by utilising a molecular charge contact method. However, these previously reported systems have a low detection limit (0.4 mM of biotin and 16.7 *μ*M of streptavidin and 2.3 $\mathrm{\mu M}$ of streptavidin, respectively) and require complicated fabrication. Although Castro, David, et al. have presented a two-phase microfluidic system which provides a user-friendly interface and simple fabrication solution, it has the relatively low detection limit, 100 ng/mL.

In this work, we introduce a T-shaped microfluidic device with a rotationally focused flow (RFF) method, enhancing the detection probable limit of a label-free optical biosensor without additional instruments even in a low concentration environment. Two fluids with different density are used to induce reduced boundary layer thickness without increasing pressure drop. For demonstrating the RFF method, three validations have been performed; simulations using Computational Fluid Dynamics (CFD) ACE^+^, a flow experiment with fluorescent beads, and a quantitative measurement of the enhanced detection limit with label-free silicon microring resonator sensors.

## Working principle of rotaional focused flow (RFF)

**Figure 1:**
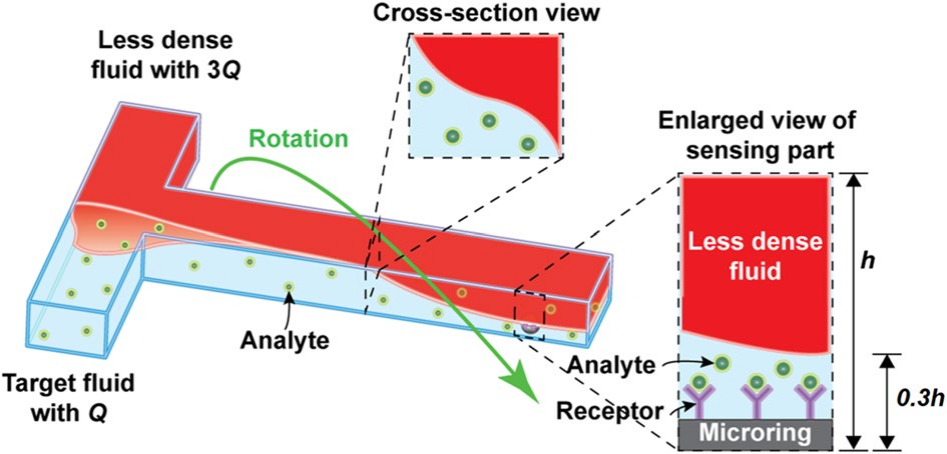
Schematic diagram of the RFF method’s concept. An injected target fluid is rotated by adding a less-dense fluid faster than the target fluid. In cross-section views at various locations, flow-twisting is observed. Injected analytes are rotationally focused near immobilised receptors onto a microring resonator by the relationship between flow rate and dynamic viscosity of a target fluid and a less-dense fluid. For example, if the volumetric flow rate of less-dense and target fluids is 3*Q*: *Q* and the dynamic viscosity of less-dense and target fluids are 5:4 in Eq. (1), the height of the target fluids is reduced to 0.3 of the height of a microchannel.

Figure 1 represents the schematic diagram of the RFF method. The fluidic channel is simple T-shaped, where two fluids with different density are introduced from two short ends and are flowed through the main channel to another end. When a target fluid is injected from one of the inlets while a less-dense fluid is pumped from the other inlet, the self-rotational flow is formed. It results in a reduction of the distance between the target and immobilised probe molecules on the sensor surface. Due to microfluidics characteristics, laminar flow with strong surface tension is expected in the rotating stream. It also enables target molecules to be focused vertically to the bottom with a low diffusion rate.

Hydrodynamically, the target biomolecules are focused near at sensors’ surface. It is to achieve a higher binding probability with immobilised probe molecules. Therefore, a faster and less-dense fluid (than the target fluids) is injected to increase the volumetric fraction ratio of the less-dense fluid. This phenomenon results in enhancement of the binding probability, as shown to the enlarged view of a sensing part in Fig. 1. The height of target fluids in RFF can be obtained by modifying an equation from Nguyen et al*.*^39^

1 $$H_{R} = \frac{{Q_{T} \mu_{L} }}{{Q_{L} \mu_{T} + Q_{T} \mu_{L} }}H_{C} ,$$

where *H*_*R*_, *Q*_*T*_, *μ*_*T*_, *Q*_*L*_, *μ*_*L*_, and *H*_*C*_ are the height of target fluids in RFF*,* the volumetric flow rate of target fluids, the dynamic viscosity of target fluids, the volumetric flow rate of less-dense fluids, the dynamic viscosity of less-dense fluids, and the height of a microchannel respectively. The fluids rotation is completed at the sensor position, and laminar flow exists. When the rotation is done, the heights can be deduced with fluids and injection properties based on Eq. (1).

Also the entrance length (*L*_*e*_) can be considered where in Laminar flow *L*_*e*_ is 0.06*R_e_*D_h_. For our system Reynolds number (*Re*) much less than 1 and D_h_ is 200 μm. The sensor is located around at 4.8 mm, and *L*_*e*_ for our system is much smaller than the sensor position. From this fact, we can see that the rotation is completed away before reaching the sensor location.

For our experiment and simulation setting, we have fixed the ratio 3:1 between *Q*_*L*_ and *Q*_*T*_ and 5:4 between *μ*_*L*_ and *μ*_*T*_ for ethanol and target fluids diluted by water, respectively. Therefore, *H*_*R*_ is reduced to 0.3 of *H*_*c*_ by adding a less-dense liquid with three times faster flow rate than target fluids based on Eq. (1).

## Method

### Computational fluid dynamics (CFD) simulation

Pattern forecast of biomolecules is essential as controlling biomolecules affects the binding probability directly. Notably, the biomolecules in microfluidic devices are more easily controlled with the laminar flow due to the high surface tension^27,40^. Fluidic simulations have been performed using CFD ACE + software (a commercial computational fluid dynamics solver, CFD Research Corporation, AL, USA). The software solution provides geometry, grid generation, solving, data visualisation. In our simulation, we utilised two representative modules in the software; “Two-fluid” and “Spray.” The “Two-fluid” was chosen to apply two independent fluids with different densities, and the “Spray” was used to assign particles as target biomolecules. For the CFD simulation, a microchannel design with 300 µm of width, 200 µm of height, and 4800 µm of length was chosen. The 200 µm height was fixed as the maximum height of microchannel fabricated by UV-lithography^41^. The 300 µm width and 4800 µm length were determined considering suitable aspect ratio (0.5–1) for the stable generation of hydrodynamic focusing^42^ and exiting silicon microring chip dimensions, respectively. Some details on the simulation parameter include diffusion coefficient of analyte, density & viscosity of target fluid, and density & viscosity of ethanol which are 1e-04 [mm2/s], ρ = 1040 kg/m^3^, μ = 0.000855 Pa s, and ρ = 789 kg/m^3^, μ = 1.04 Pa s respectively.

### Material selection

A Poly (dimethylsiloxane) (PDMS) kit was purchased from Dow Corning (USA). Distilled (DI) water and phosphate-buffered saline (1X PBS) were purchased from Invitrogen (Carlsbad, CA, USA). 97% trichloro (3, 3, 3,-trifluoropropyl) silane was purchased from Fluka (Buchs, Switzerland). SU-8 2100 and SU-8 developer were purchased from Microchem (Newton, MA, USA). 3-aminopropyltriethoxysilane (APTES), bovine serum albumin (BSA), Isopropyl alcohol (IPA), 99.8% ethanol, denatured ethanol, and latex bead type of 1 µm amine-modified polystyrene with fluorescent red were purchased from Sigma-Aldrich (St. Louis, MO, USA). Immunopure streptavidin and EZ-Link NHS-PEG_4_-biotin were purchased from Thermo Scientific Pierce (Singapore). Biotin anti-human TNF-α was purchased from BioLegend (San Diego, CA, USA). Other chemicals were analytical reagent grade and were used as received. All samples and buffers were prepared using DI water and PBS.

### Design and fabrication

A simple T-shaped microchannel which has the same dimension with the CFD simulation was printed onto a film photomask. PDMS microfluidic channels were built by PDMS replica molding from SU-8/silicon masters that were fabricated by the conventional UV-photolithographic process using the film mask. For 200 µm of target thickness, SU-8 2100 of a photoresist was poured onto an 8-inch silicon substrate and spin-coated with the spin speed of 1400 rpm for 60 s. The spin-coated photoresist was then baked at 65 °C for 7 min and 95 °C for 40 min. The baked layer covered with the designed photomask was then exposed to a 315 mJ/cm^2^ dose of the UV light. In a post-exposure baking process, the transferred photoresist was then baked on a hotplate at 65 °C for 5 min and 95 °C for 14 min. Finally, through a development process for 17 min using a SU-8 developer and rinse process by IPA and DI water, a SU-8 master for the replication of microfluidic devices was realised.

PDMS is the most common polymer for the fabrication of microfluidic devices because of those advantages such as low-cost and straightforward replication process^17^. The fabricated SU-8/silicon master was coated for the silanisation using 97% trichlorosilane for 30 min. This process is to prevent the adhesion between the master and PDMS replicas^43^. The mixture of the PDMS prepolymer (sylgard 184, part A) and the curing agent (sylgard 184, part B) with a 10:1 weight ratio was evenly stirred for 10 min using a glass rod. The PDMS mixture was then poured onto a SU-8/silicon master. The PDMS mixture on the master was then degassed inside a vacuum desiccator and cured for one hour at 80 °C in a convection oven.

**Figure 2:**
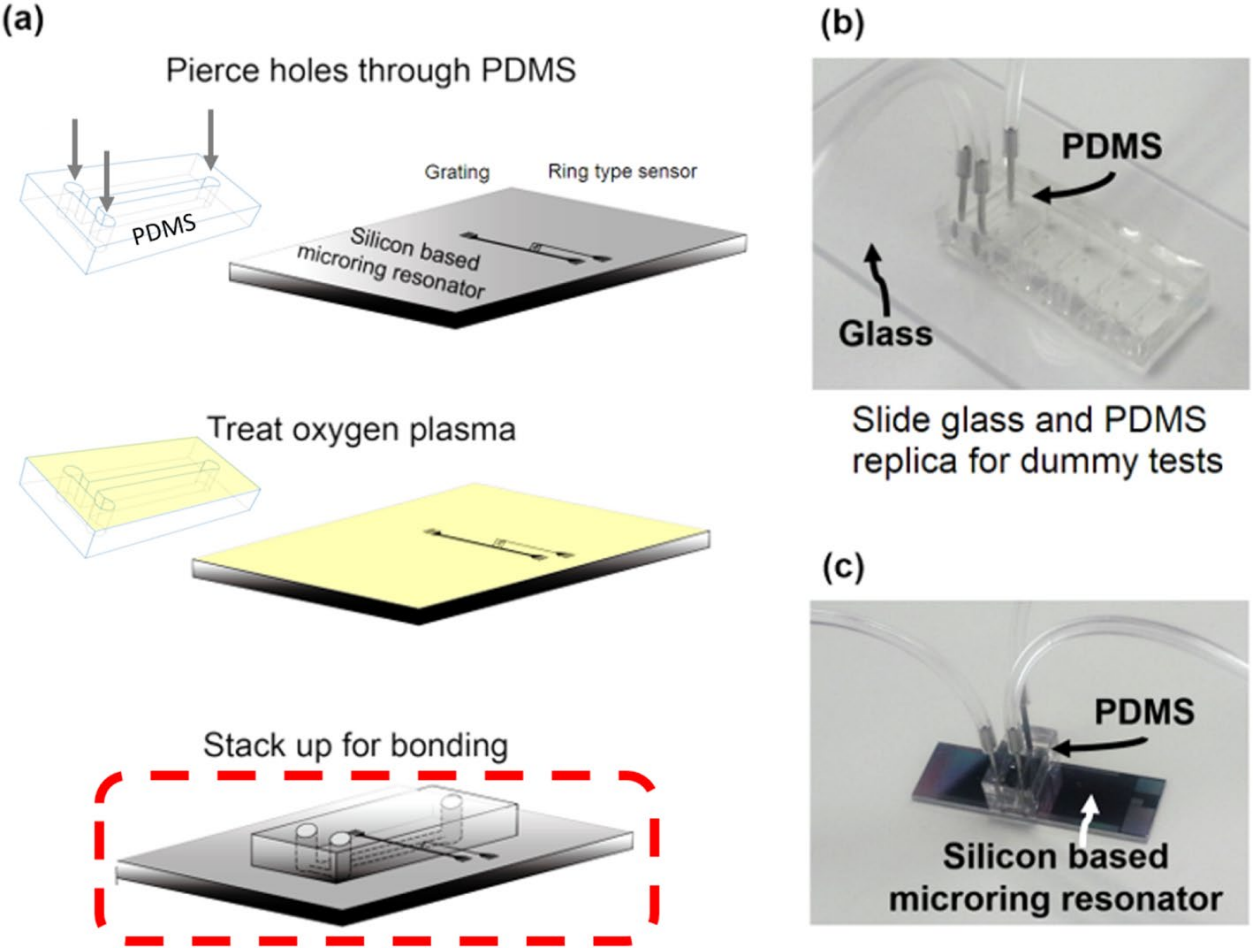
(**a**) Schematic diagram of fabrication of a silicon-based label-free optical biosensor. For bonding, O_2_ plasma was used. Images of; (**b**) fabricated PDMS/glass microfluidic device by replacing the silicon-based microring resonator with the glass for dummy test and (**c**) the fabricated silicon-based label-free optical biosensor.

There are two experiments; dummy and real tests. Hence bonding processes of molded PMDS replicas have been performed twice, one with sliding glass and the other with a silicon-based microring resonator sensor chip (Fig. 2). The silicon-based microring resonator has been fabricated with 220 nm thickness of SOI wafers with 2 μm of buried oxide (BOX) layer in our previous work^20,43,44^. In brief, one linear input waveguide is connected with four microrings, and each microring has a corresponding output waveguide. One of the microrings’s output is used as a reference while the 3 remaining microrings’ are collected through a vertical grating coupler (Fig. 2a).

The dimension of racetrack-shaped microrings is 5 μm of the radius with 2.042 of coupling length. Besides, the width of waveguides is 500 nm, and the gap between a linear waveguide and a microring is 220 nm. A PDMS replica was pierced using a steel punch to connect tubes at inlet and outlet positions before a bonding process. Prepared PDMS replica and slide glass (or silicon-based microring resonator) surfaces were washed with running IPA and DI water, followed by drying under a nitrogen stream and by heating in a convection oven. They were then treated with the oxygen plasma to activate the hydroxyl group for the adhesion using the NT-2 plasma cleaning system (Anatech Ltd., CA) under 70 W of a treating power in 60 sccm of O_2_ flow rate for 50 s. The treated PDMS replica was bonded with the sliding glass for the verification of the RFF. Also, the microchannel in the treated PDMS replica was aligned and bonded to the microring sensor chip guided by an inverted microscope with CCD camera (Fig. 2a). Finally, two types of microfluidic devices (Fig. 2b,c) have built after a hardening process in a convection oven at 150 °C for 30 min.

### Flow experiment

To observe the RFF generation in the fabricated PDMS microchannel, a fluorescence experiment was performed using fluorescent polystyrene beads in the PBS solution. The solution of fluorescent polystyrene beads and ethanol were prepared in 1 mL of syringes separately and injected by two syringe pumps (Scientific Inc., model KD 200 series, USA) for independent control of fluids. A bottom view of the microchannel was taken using an inverted microscope (Olympus, model IX71, Japan) integrated with a CCD camera (Q imaging, model Rolera-XR, USA) (Fig. 4).

### Surface chemistry

For the non-specific binding of streptavidin, 3-aminopropyltriethoxysilane (APTES), NHS-PEG_4_-biotin, and bovine serum albumin (BSA) were treated using syringe pumps. The integrated PDMS/silicon-based microring resonator device was firstly treated with oxygen plasma during the bonding process. It was then treated by injecting a solution of 2% APTES in a mixture of ethanol/H_2_O (0.95/0.05, v/v) via the left inlet using a syringe pump for 2 h, followed by thorough rinsing with ethanol. It was then treated through injection of 0.25 mg/mL solution of NHS-PEG_4_-biotin in DI water for 1 h and rinsed with DI water in the same method. Also, 0.1% of BSA in PBS was injected into the left inlet for 30 min to prevent non-specific binding at the surface of a silicon-based microring resonator and rinsed with PBS.

### Quantitative performance tests

For the quantitative characterisation of the fabricated silicon-based label-free optical biosensors, wavelength shifts by biotin-streptavidin interaction were measured with different flow methods. Due to the highest non-covalent binding affinity (${K}_{D}={10}^{-15}\mathrm{M})$, biotin-streptavidin interaction is frequently used to characterise the binding performance of biosensors^18,45^. The equipment set-up with a LabVIEW program for the optical measurement and the optical sensing principle was referenced from our previous works^44,46,47^.

## Results and discussion

### CFD simulation

**Figure 3:**
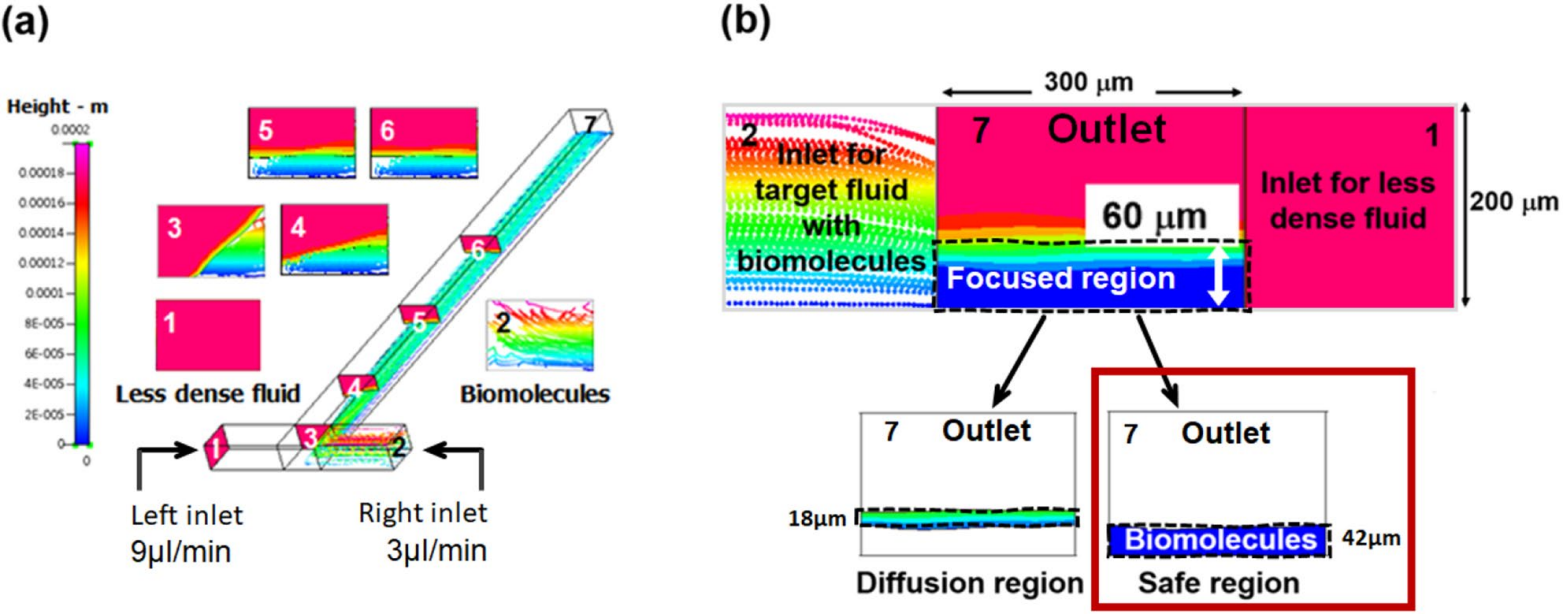
CFD simulation results investigations for; (**a**) the RFF generation by observing target molecule’s behaviors with numbered cross-section views at the left inlet (1), the right inlet (2), the main channel entrance (3), 1 mm (4), 2 mm (5), 3 mm (6), 4.8 mm (7) distant from the entry, (**b**) the denaturation safety analysis of target molecules by ethanol at an outlet of a microchannel (at 7—where the microring sensor is attached) with 60 μm of a focused region, 18 μm of a diffusion region and 42 μm of the safe region thicknesses. The RFF was formed by injecting 3 μL/min of PBS, including polystyrene beads (ball typed dots) as a target fluid and 9 μL/min of ethanol as a less-dense fluid for the CFD simulation. Both fluids are flowing continuously.

Figure 3 represents the CFD simulation results of the RFF. The legend, height, indicates vertical locations of target molecules focused under diffusion layers. For the precise observation of biomolecule’s behaviors, both of injected fluids were disabled in the result view except infused polystyrene beads, as shown in Fig. 3a. In cross-section views at various locations, rotation processes and vertically focused polystyrene beads near the bottom side of a microchannel were observed. Ethanol was added three times more than polystyrene beads solution. Rotational flow stream and vertically focused hydrodynamic biomolecules were found through the cross-section views. The RFF was already achieved at the beginning of a microchannel outlet (between positions 4 and 5) with less-dense fluids height ~ 140 μm and focused fluids height of ~ 60 μm, which agrees with Eq. (1)^42^.

For an ideal choice of less-dense fluid, the density difference between two fluids must be large enough to ensure rapid fluid rotation to minimise diffusion between them. In this project, therefore, ethanol was selected (Δρ = 251 kg/m^3^, an ethanol density of 789 kg/m^3^) and the viscosity of the target fluid (PSB + Polystyrene bead) was 1040 kg/m^3^ where the density of Polystyrene bead is 1050 kg/m3. One of the major concerns for using ethanol could be the probability of biomolecules denaturation^48^ when mixing two fluids. However, in a typical microfluidic configuration, mixing is unlikely to happen due to negligible Reynolds number except for a little diffusion^27,49^. The Reynolds number (*Re*) can be considered as $$Re=\frac{D*V*\rho }{\mu }$$, where D, V, ρ, and μ are dimension, velocity, density, and viscosity, respectively. In specific to our system, the ρ and μ values are the same as our simulation values given for the simulation, and micro (10^–6^) can represent our experimental dimension. It leads us to have $$Re=\frac{{10}^{-6}*V*1040}{0.000855}\approx 1.77*V.$$ Based on our experiment and simulation velocity of fluids (3 μL/min, 9 μL/min), we can confirm that our Reynolds number is much less than 1, satisfying the small Reynolds condition microfluidic environment. Our system method employed both Laminar flow and self-rotating. Small Reynolds number ensures Laminar flow domination which does not to cause diffusion. Fluids with different densities enable self-rotating in microchannel^42^, and it helps to focus the particles on the sensor surface (i.e., Fig. 3(b) safe region). Therefore, the denaturation of input biomolecules can be avoided by controlling the diffusion layer thickness consistency. The CFD simulation was carried out to confirm this phenomenon. Figure 3b represents the backside view (position 7) of a microchannel in a CFD simulation to investigate the diffusion layer thickness and a safe region from the denaturation. In this figure, red block, blue block, and ball typed dots indicate ethanol, PBS, and polystyrene beads, respectively. The simulation results show two distinct regions within the focused region of RFF; a diffusion region and a safety region where biomolecules do not mix with ethanol. The diffusion region forms beyond 42 μm above the bottom surface, which provides enough distance to prevent the diffusion of denatured molecules to the sensor surface. Therefore, the RFF method using ethanol is suitable to be used with our microfluidic device, and the method can enhance their binding probability by reducing a distance between target molecules and immobilised probe molecules.

### Flow experiment using fluorescent beads

**Figure 4:**
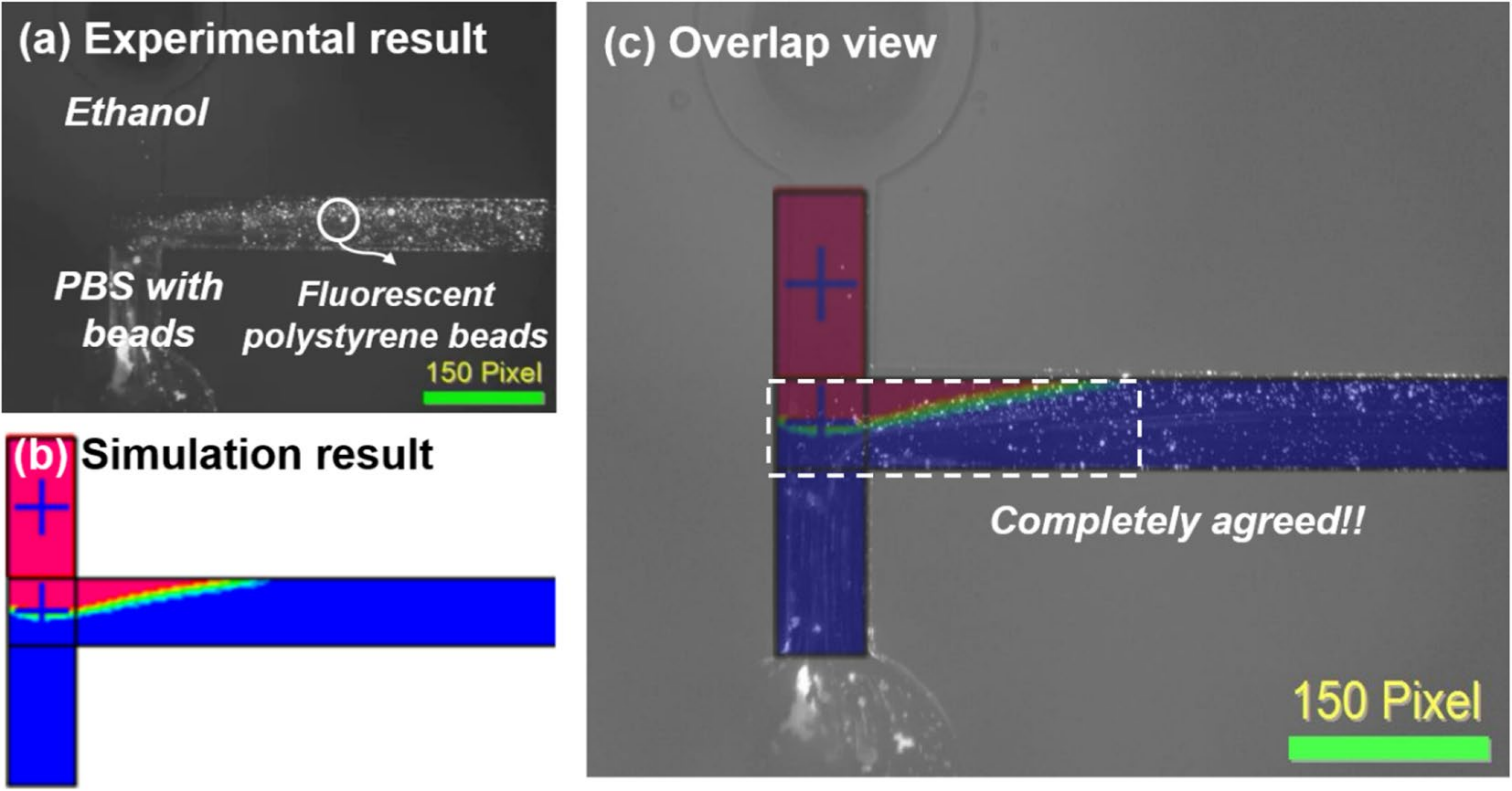
A bottom view of microchannels taken by an inverted microscope; (**a**) the experimental result of a flow pattern which was made by injecting 3 μL/min of PBS including fluorescent polystyrene beads with 9 μL/min of ethanol, (**b**) simulation result in the same geometric and flow conditions to the flow experiment in (**a**), (**c**) an overlapped image of (**a**) and (**b**).

In this flow experiment, 10 μg/mL solution of fluorescent polystyrene beads in PBS was injected to the right inlet with 3 μL/min of volumetric flow rate, and ethanol was injected to the left inlet with 9 μL/min of volumetric flow rate. As shown in Fig. 4a, the injected fluorescent polystyrene beads from the right inlet were flowed with becoming more extensive to the left side of a microchannel. According to a flow pattern of the injected fluorescent polystyrene beads, the flow can be perceived as a rotation flow by the density difference between the solution of fluorescent polystyrene beads and ethanol. Also, the injected fluorescent polystyrene beads were not escaped from the flow boundary between the two fluids over time, as shown in Fig. 4a. Figure 4b shows the bottom view of CFD simulation results in 300 μm of width, 200 μm of height, and 4800 μm of length device design with the same flow condition as a flow experiment. For more accurate verification, a flow pattern of the fluorescent polystyrene beads in the experimental result (Fig. 4a) was overlaid with the CFD simulation result (Fig. 4b). Remarkably, both images are perfectly aligned, which confirms the generation of the RFF, as shown in Fig. 4c.

### Binding performance

The binding performance was investigated using a silicon-based microring sensor. The microring sensor is a label-free refractive index sensor by taking advantage of the evanescent filed present near the ring resonator surface^50^. Its resonant wavelength, *λ,* is directly related to the effective refractive index of the surrounding area and the antibody-antigen interaction can be monitored by measuring the shift in the resonant wavelength, Δ*λ*^44,46,51^.

**Figure 5:**
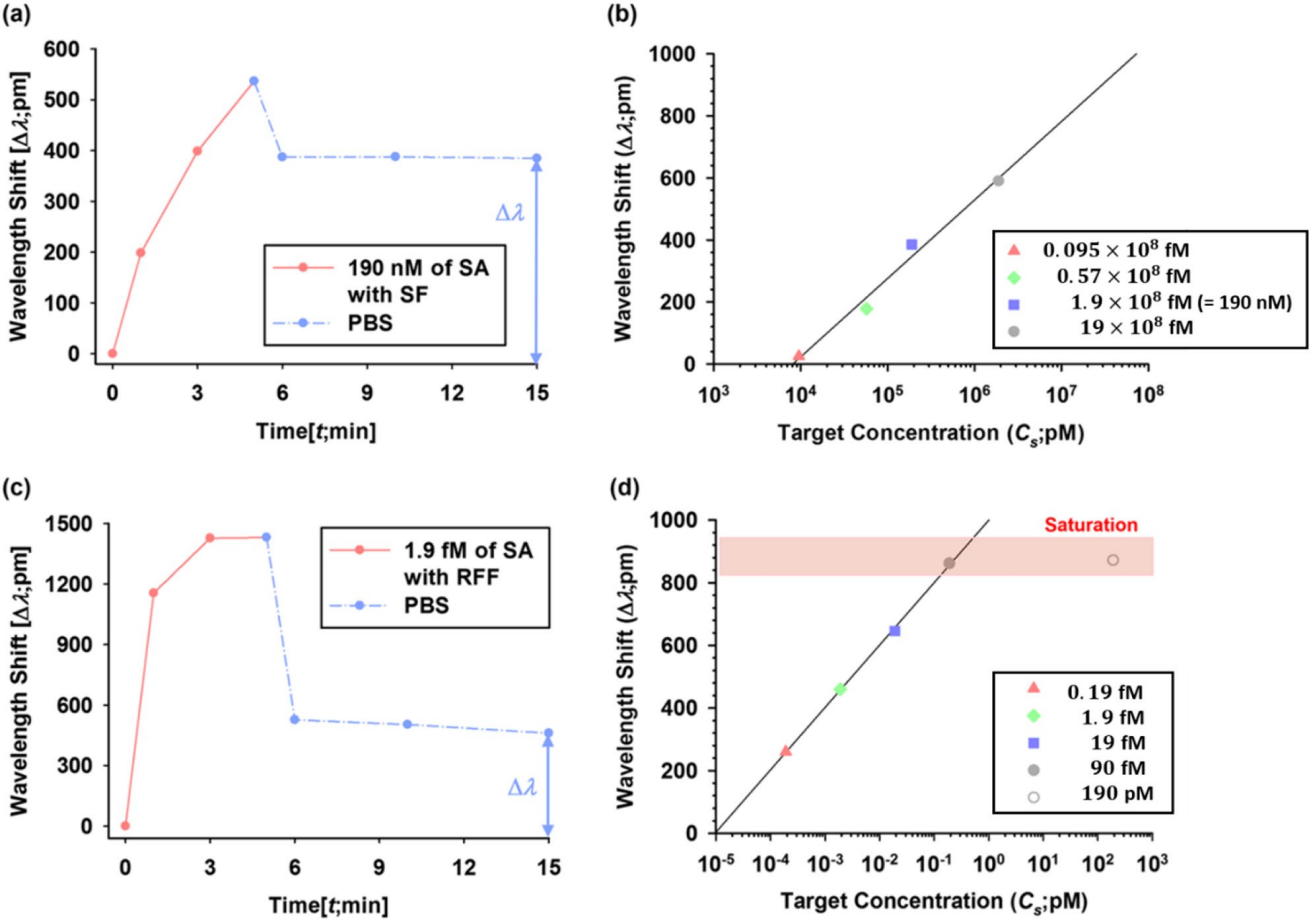
Binding curves of; (**a**) wavelength shifts of 190 nM Streptavidin solution injected by the Single Flow (SF) method, (**b**) the resonance shift values by SF method at 15 min (10 min after PBS washing) plotted against the different concentration of Streptavidin solution ranging from 9.5 nM (= $0.095\times {10}^{8}$fM) to 1900 nM (= $19\times {10}^{8}$fM) (R^2^ = 0.9838), (**c**) wavelength shifts of 1.9 fM Streptavidin solution injected by the RFF method and (**d**) the resonance shift values by RFF method at 15 min (10 min after PBS washing) plotted against the various concentration of Streptavidin solution ranging from 0.19 fM to 190 pM (R^2^ = 0.9992). For (**a**) and (**c**) the dotted blue lines are plotting wavelength shift after PBS washing. This process is required for ensuring single layer molecule binding on the sensor surface.

The binding performance of the RFF method was compared with that of the single flow (SF) method using Streptavidin–biotin binding as an antigen–antibody model case. The SF is a typical flow method that target fluids are injected solo into a microchannel. The streptavidin solution was injected into a microchannel without using a less-dense fluid. The binding curve of 190 nM of Streptavidin solution injected by the SF method is shown in Fig. 5a. The microchannel was first filled with PBS to obtain the baseline then the Streptavidin solution was injected at 3 μL/min of a volumetric flow rate for 5 min followed by PBS washing for 10 min to remove any non-specific binding. The binding curves of various Streptavidin solutions ranging from 9.5 nM to 1900 nM concentrations were obtained. And the resonance shift values at 15 min (10 min after PBS washing) are plotted against the concentration of Streptavidin solution in Fig. 5b. The resonant wavelength shifts at 15 min were 25, 178, 385, and 590 pm corresponding to 9.5, 57, 190, and 1900 nM of Streptavidin solutions, respectively. The graph shows a logarithmic growth, with a correlation coefficient of the least square (R^2^ = 0.9838).

In the RFF method experiment, ethanol was added as a less-dense fluid to a microchannel. Figure 5c represents a binding curve of 1.9 fM of Streptavidin solution injected by the RFF method. The microchannel was first filled with PBS to obtain the baseline; Streptavidin analytes were then injected with 3 μL/min of a volumetric flow rate, and ethanol was injected with 9 μL/min of a volumetric flow rate. After 5 min, the entire microchannel was washed with PBS for 10 min for removing non-specific binding. More Streptavidin molecules near the sensor surface were observed from the RFF method than the SF method.

The molecules could accumulate near the sensor surface, and this could generate a large amount of non-specific bindings and result in a significant drop-off in the wavelength shifts during PBS washing. The measured wavelength shifts at 15 min (10 min after PBS washing) by the biotin-Streptavidin interaction using the RFF method in various concentrations of Streptavidin solutions ranging from 0.19 fM to 190 fM were plotted as shown in Fig. 5d. The resonant wavelength shifts were 260, 460, 646, and 862 pm corresponding to 0.19, 1.9, 19, and 190 fM of Streptavidin solutions, respectively. Similar to the SF method, the conjugations tendency grows logarithmically with a correlation coefficient in the least square method (R^2^ = 0.9992). One may read from the stable wavelength shift values from (a) and (c), they are similar (around 400 pm). Notice that results were measured from the SF method with 1.9*108fM while RFF with 1.9fM concentration. Remarkably, the sensitivity of the microring sensor with the RFF method was improved by 8-order of magnitudes compared to the SF method. Note that the purpose of Fig. 5 is not comparing each sensor’s performance to the saturation value. It is rather to examine the sensing possible limit varying concentration. The essential focus was on “detection possibility,” as long as the binding quantity is reasonable to cause wavelength shift. The results can benefit early diagnosis where samples’ concentration is relatively low. When concentration is very low, probability to molecule binding is low compared to the single flow. In usual case, with low concentration, the molecule does not reach to the sensor surface. But with the RFF system, even with a diluted sample (low concentration), the rotational force helped to position the molecule near to the sensor surface, hence increase binding probability.

Binding curves for both methods were well fitted with the Langmuir model with correlation coefficients from plotted graphs. For the Langmuir model, the apparent dissociation constant (*K*_*d*_) is defined as^44^

2 $$\Delta \lambda = \lambda_{\max } \frac{{C_{s} }}{{K_{d} + C_{s} }},$$

where Δ*λ* is the measured resonant wavelength shift, *λ*_max_ is the maximum wavelength shift for bound streptavidin to immobilised biotin onto a microring resonator. And *C*_*s*_ and *K*_*d*_ are the concentration of Streptavidin solution and the apparent dissociation constant, respectively.

In these experiments, over 190 fM of Streptavidin concentrations, wavelength shifts were varied from 800 to 920 pm. According to the data, we can estimate *λ*_max_ as 860 pm. *K*_*d*_ between Streptavidin molecules and immobilised biotins onto a microring resonator were calculated using Eq. (2) to be 4.12 (± 0.53) × 10^–7^ M for the SF method and 2.82 (± 3.12) × 10^–15^ M for the RFF method. This result shows the significant enhancement of the sensitivity and detection limits by the RFF method.

### Biological activity test

**Figure 6:**
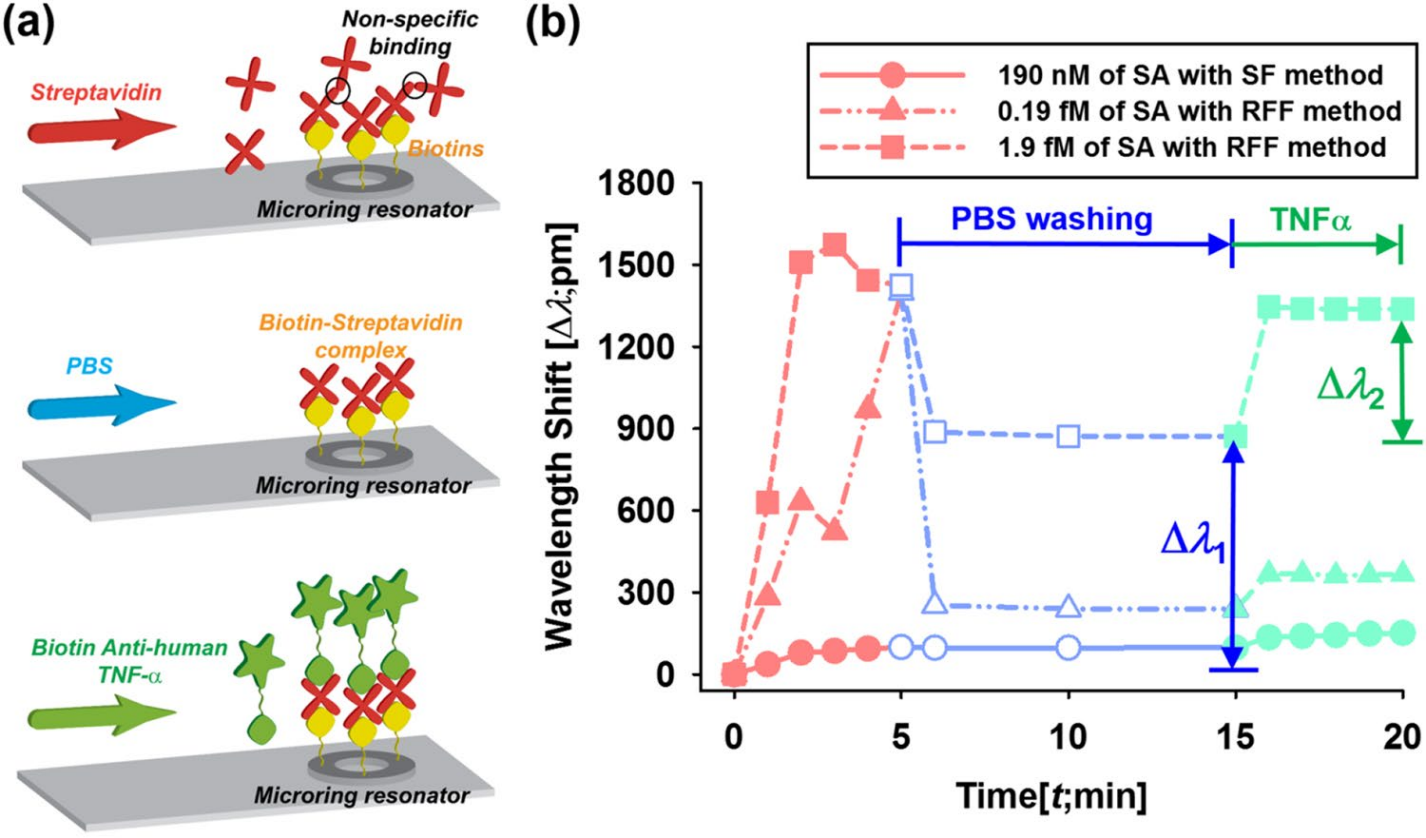
(**a**) Schematic diagram of experimental procedures to investigate the biological activity of conjugated Streptavidin to immobilised biotins onto a microring resonator by injecting Streptavidin solution, PBS and biotin-TNFα solution and (**b**) the binding curves of the biotin-Streptavidin interaction using various concentrations of Streptavidin solutions (red line; 0–5 min) with different flow methods (solid line; 190 nM Streptavidin solution with SF method, dot double-dashed line; 0.19 fM Streptavidin solution with RFF method and dashed line; 1.9 fM Streptavidin solution with the RFF method) followed by PBS washing (blue line; 5–15 min) and subsequent interaction between bound Streptavidin and biotin-TNFα (green line; 15–20 min).

In order to confirm that enhanced apparent sensitivity of RFF method^44^ is based on real increase in the sensitivity not due to other factors including non-specific binding of denatured protein clusters, we investigated the biological activity of bound Streptavidin molecules using biotinylated anti-human TNF- α (biotin-TNFα) as a secondary probe. Figure 6 shows binding procedures and the binding curves of the biotin-Streptavidin interaction using various concentrations of Streptavidin solutions (red line) with different flow methods followed by PBS washing (blue line) and subsequent interaction between bound Streptavidin and biotin-TNFα (green line). The schematic of the detailed binding procedures is shown in Fig. 6a. The binding curves for 190 nM of Streptavidin solution using the SF method, 0.19 fM and 1.9 fM of Streptavidin solution using RFF method are shown in Fig. 6b. Numerical values of wavelength shifts at 5 min (after Streptavidin binding), 15 min (after washing) and 20 min (after biotin- TNFα binding) are listed in Table 1. The wavelength shifts at 15 min by the biotin-Streptavidin interaction were 98, 239.4 and 872.7 pm for 190 nM of Streptavidin/the SF method, 0.19 fM of Streptavidin/RFF method and 1.9 fM of Streptavidin/RFF method, respectively. After injecting 10 μg/mL of biotin-TNFα for 5 min, the resonant wavelengths were shifted further as 53.5, 127.4 and 465.3 pm, respectively due to the binding between bound Streptavidin and biotin- TNFα. High concentration of biotin-TNFα was used to ensure all bound Streptavidin molecules are fully conjugated with biotin-TNFα. In principle, the resonant wavelength is proportional to the mass change of analytes bound to receptors onto the sensor surface^44^. In this experiment, we used 26 kD of biotin-TNFα and 53 kD of Streptavidin. Based on the molecular weight ratio, the ratios of wavelength shifts by the Streptavidin-TNF-α with the wavelength shifts by the biotin-Streptavidin interaction in cases of 190 nM of Streptavidin/SF method, 0.19 fM of Streptavidin/RFF method and 1.9 fM of Streptavidin/RFF method were 0.54, 0.53 and 0.53, respectively (Table 1). This result clearly shows that all bound Streptavidin molecules using RFF methods are biologically active and verifies the enhanced sensitivity induced by RFF method.

**Table 1:** Measured values of wavelength shifts by various combinations of Streptavidin solutions with flow methods in each testing procedure and calculated ratios by Δλ2/Δλ1 as described in Fig. 6b.

Flow types	~ 5 min		~ 15 min		~ 20 min		Ratio (Δ*λ*_2_/Δ*λ*_1_)
	Solution	Δ*λ*	Solution	Δ*λ*_1_	Solution	Δ*λ*_2_	
SF	190 nM of SA	99.7 pm	PBS	98 pm	190 nM of biotin-TNFα	53.5 pm	0.54
RFF	0.19 fM of SA	1400 pm	PBS	239.4 pm	190 nM of biotin-TNFα	127.4 pm	0.53
RFF	1.9 fM of SA	1425.7 pm	PBS	872.7 pm	190 nM of biotin-TNFα	465.3 pm	0.53

### Potential migration of biomolecules by Dean vortex

As we discussed earlier, drastically enhanced sensitivity by the RFF method is beyond expectation. Besides a simple channel height reduction effect, the secondary flow effect has been considered to explain the target analyte flux increment further.

**Figure 7:**
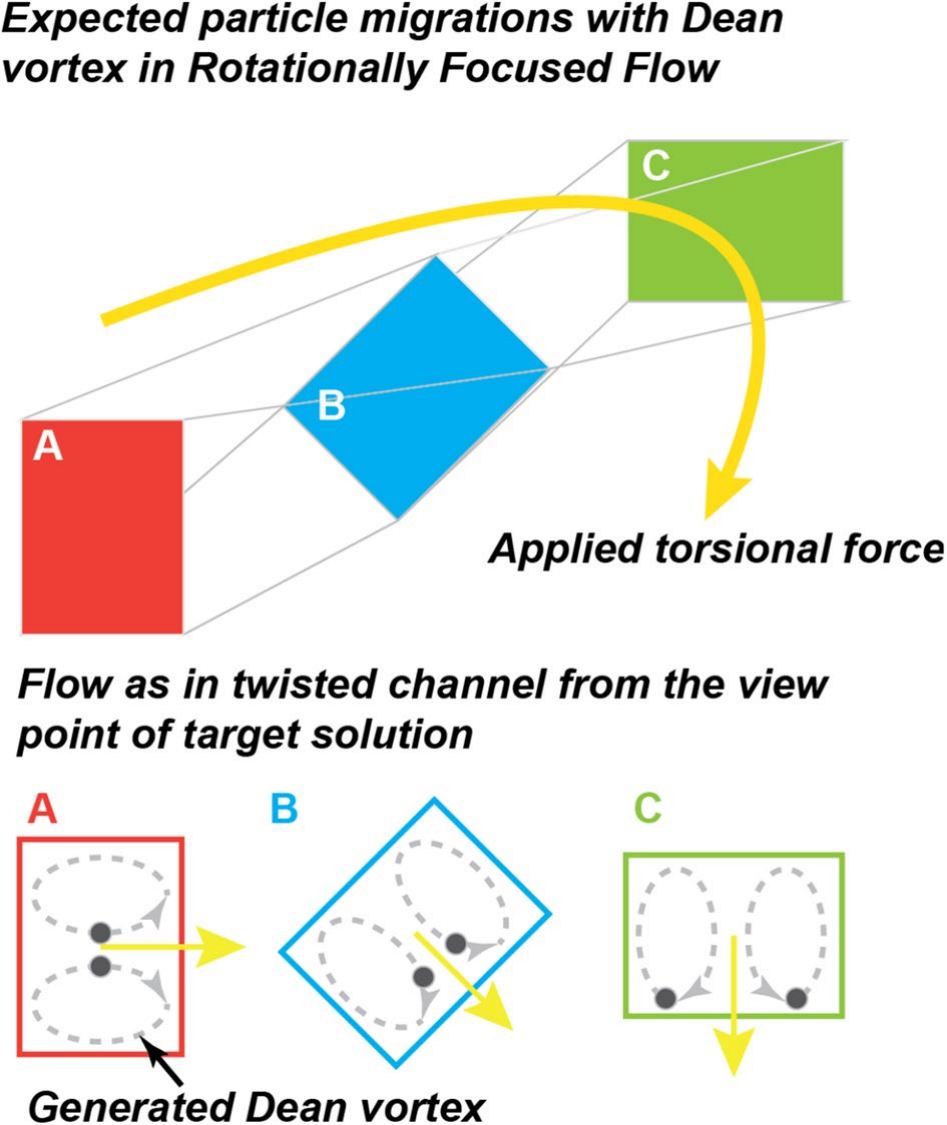
Schematic diagram of Dean vortex in RFF at target fluid point of view. Particle migrations by the Dean vortex are illustrated based on microchannel positions in Fig. 3a (**A**: position 2–3, position **B**: 3–5, position **C**: 5–7).

Among various secondary flows, the transverse motion of particles by the Dean vortex is well-known to have a significant effect on the enhancement of the sensitivity^52–55^. Generally, particle migrations in laminar flow are changed when fluids flow in a curved, bent or twisted structures. Inside the structures, particles are forced from the centre to the outer side by centrifugal force or torsional force with a double symmetric vortex upward and downward^52–54,56^. Figure 7 illustrates the generated vortex and how migrations of particles would happen in the RFF method. In the clockwise rotating region, the target fluid with biomolecules is forced according to the direction of rotation. In this case, the transverse vortex motion of biomolecules is expected as in a twisted-channel without passing through the interface of two fluids based on high surface tension and diffusion-limited transportation of microfluidic characteristics as shown in Fig. 7. It could result in the active migration of biomolecules closer to the sensor surface. Therefore, Dean vortex is one explanation for the drastically enhanced sensitivity of the RFF method. However, further investigation should be carried out in the future to seek theoretical and experimental proof.

## Conclusion and future work

We presented the RFF method for enhancing the sensitivity of label-free optical biosensors by adding a less-dense fluid horizontally. The hydrodynamically focused rate was controlled by changing the volumetric fraction ratio between the target fluid and the less-dense fluid. These phenomena were resulting in the enhancement of the binding. For the investigation, microfluidic devices were designed with the RFF method, and CFD simulations, flow experiments, and quantitative measurements were performed.

The formation of RFF and behaviours of biomolecules inside a simple T-shaped microchannel were investigated in CFD simulations and fluorescent bead experiments. The CFD simulation confirms the hydrodynamic focusing of target fluids (3/10 of the channel height) when the volumetric flow rate ratio of less-dense fluid, ethanol, to the target fluid is fixed to 3:1 which agrees with theoretical expectation.

For the quantitative characterisation of binding performances, optical measurements of silicon-based label-free optical biosensors were carried out using biotin-streptavidin as a model antigen–antibody system with different flow methods. The results showed drastic improvement (8-order of magnitudes) in the sensitivity of the biotin-modified microring sensor in the RFF method compared to the SF method. For both methods, the conjugation tendency (between streptavidin and immobilised biotin) was logarithmically proportional to Streptavidin solutions’ concentrations. The apparent dissociation constants obtained from the Langmuir models were to be 4.12 (± 0.53) × 10^–7^ M for the SF method and 2.82 (± 3.12) × 10^–15^ M for the RFF method. We conducted a series of experiments including bioactivity testing and comparison with the VFF method, which has the height reduction effect purely and verified the real effect of the RFF method for significant improvement in the sensitivity of the optical sensor. Also, a low concentration rate with 0.19 fM Streptavidin solution was tested with promising detection results, which could have been lowered further to check the lowest possible concentration limit.

Along with the channel height reduction effect, the transverse motion of particles by the Dean vortex can explain one of the dominant impacts on the sensitivity enhancement. The RFF method offers a simple and effective way to enhance the sensitivity of the label-free optical sensor. It can be widely applied to any biosensor without requiring additional instruments.

Among current diagnostic tests for COVID-19, nucleic acid testing (e.g., RT-PCR) is the most predominantly used methods^57,58^. However, its time consuming and complicated process, including the preparation of viral RNA, the reverse transcription and amplification steps signify the need for the development of rapid and sensitive POC diagnostics for SARS-CoV-2, the viral aetiology of COVID-19^35^. Moreover, a recent study highlights the early-stage COVID-19 detection with protein-biomarker based testing^34^. The early-stage diagnosis can help reduce and slow-down the risk of viral transmission. Therefore, our RFF method, integrated with widely used microring resonators based on the antigen–antibody interaction, provides an approachable concept to develop essential tools for early detection of COVID-19 with shortening process time. It is a microfluidic-based sensor system which requires a minimal amount of sample. Although we used Streptavidin–biotin binding as a model case for antigen–antibody, further study for detection of COVID-19 using the RFF method with SARS-CoV-2 antigen should be investigated so that the suggested method can be quickly implemented in the current COVID-19 pandemic.

Researchers and industries worldwide have been competing and collaborating to achieve a significant breakthrough to combat the COVID-19. Cutting-edge methods have advanced biosensing technologies to realise ultrasensitive, ultrafast, and early detection in pandemic diseases. Although most of the attempts are not practically viable, numerous research ideas have been published regarding COVID-19 keeping limitations and challenges; the research attempt will never be enough until we conquer the COVID-19^22^.
